# Surgical management and outcomes of catamenial pneumothorax: a European multicentre real-life comparative study

**DOI:** 10.1093/icvts/ivaf129

**Published:** 2025-07-01

**Authors:** Lavinia Gatteschi, Domenico Viggiano, Rossella Indino, Laura Socci, Marco Lucchi, Maria Giovanna Mastromarino, Marcelo F Jimenez, Marta Fuentes-Gago, Giuseppe Marulli, Rosatea Quercia, Luca Voltolini, Alessandro Gonfiotti

**Affiliations:** Thoracic Surgery Unit, Careggi University Hospital, Florence, Italy; Thoracic Surgery Unit, Careggi University Hospital, Florence, Italy; Thoracic Surgery Unit, Careggi University Hospital, Florence, Italy; Department of Thoracic Surgery, Sheffield Teaching Hospitals NHS Foundation Trust, Sheffield, UK; Department of Thoracic Surgery, University Hospitals Bristol and Weston NHS Foundation Trust, Bristol, UK; Division of Thoracic Surgery, Cardiac-Thoracic and Vascular Department, University Hospital of Pisa, Pisa, Italy; Division of Thoracic Surgery, Cardiac-Thoracic and Vascular Department, University Hospital of Pisa, Pisa, Italy; Service of Thoracic Surgery, Salamanca University Hospital, Salamanca, Spain; Service of Thoracic Surgery, Salamanca University Hospital, Salamanca, Spain; Unit of Thoracic Surgery, Department of Precision and Regenerative Medicine and Ionian Area, University of Bari “Aldo Moro”, Bari, Italy; Department of Biomedical Sciences, Thoracic Surgery, Humanitas University, IRCCS Humanitas Research Hospital, Milan, Italy; Unit of Thoracic Surgery, Department of Precision and Regenerative Medicine and Ionian Area, University of Bari “Aldo Moro”, Bari, Italy; Thoracic Surgery Unit, Careggi University Hospital, Florence, Italy; Thoracic Surgery Unit, Careggi University Hospital, Florence, Italy

**Keywords:** catamenial pneumothorax, VATS, diaphragmatic surgery

## Abstract

**OBJECTIVES:**

Catamenial pneumothorax is an underdiagnosed condition, despite accounting for up to 35% of spontaneous pneumothoraces in young women. This study aims to delineate the most appropriate surgical treatment comparing a 15-year experience of five European centres.

**METHODS:**

A European multicentre retrospective cohort study was conducted. We evaluated all the spontaneous pneumothoraces occurring in women of childbearing age. We included all the cases with evidence of diaphragmatic alterations. Thirty-six patients were included and evaluated. We compared their surgical treatment, in-hospital variables and rate of recurrence.

**RESULTS:**

The surgical approach was thoracoscopic for 34 patients and open for 2. Thirty patients presented diaphragmatic involvement. According to the diaphragmatic treatment the patients were divided into three groups: prosthetic replacement (19; 15 synthetic grafts and 5 biological); surgical repair (6; 4 direct sutures and 2 stapling); and no treatment (11). All patients received pleurodesis (6 mechanical, 15 chemical and 3 a combination of these). Median follow-up was 54 months, during which 15 recurrences occurred. Despite no statistically significant difference between the treatment groups, the relapse rate slightly favoured the prosthetic group (26.3% vs 56.8%, *P* = 0.09) and direct diaphragmatic repair had a significantly higher conversion rate compared with prosthetic replacement (21% vs 100%, *P* = 0.004). Notably, none of the patients with biological mesh relapsed during the follow-up.

**CONCLUSIONS:**

Our data suggest treating all diaphragmatic defects with thorough attention. Moreover, prosthetic replacement resulted in a safe and effective procedure and biological mesh should be preferred in this setting, showing excellent postoperative and long-term results.

**CLINICAL REGISTRATION NUMBER:**

Our institutional review board granted approval and waived the requirement for specific informed consent for this retrospective analysis.

## Graphical Abstract

**Figure:**
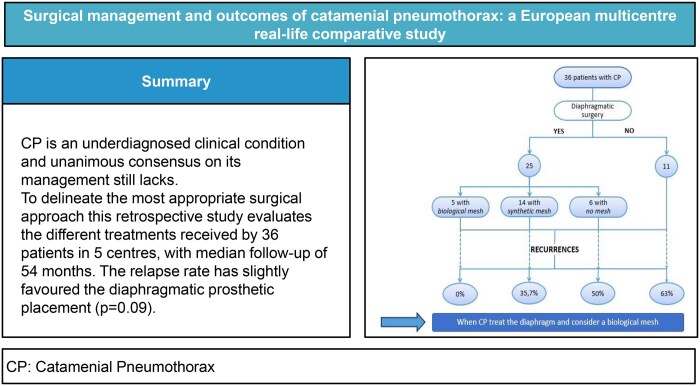

## INTRODUCTION

Catamenial pneumothorax (CP) is an intriguing clinical entity characterized by the recurrent and spontaneous accumulation of air within the pleural space, affecting women of reproductive age.

The term ‘catamenial’ derives from the Greek word ‘καταμένιος’, which means ‘monthly’, as this condition is often associated with the menstrual cycle.

Its distinct pattern of recurrence typically coincides with the so-called ‘perimenstrual period’, which ranges from the day before to 3 days after the onset of menstruation [1, 2].

In recent years the understanding of CP has evolved and it is now accepted that it accounts for a significant proportion, estimated at 20–35%, of spontaneous pneumothorax (SP) cases among women of childbearing age [3–5].

The onset of CP occurs at an older age (mean age 34–37 years) than in cases of primary SP and in the majority of the cases it is right-sided (95%) [6].

It is recognized as the most common manifestation of thoracic endometriosis (TE) syndrome, which comprises four distinct entities: CP; catamenial haemothorax; catamenial haemoptysis; and endometriotic lung nodules [4]. Nevertheless, the exact incidence of endometriosis in CP cases can vary widely, ranging from 52% to 87.5%, depending on the expertise of the authors [5].

While CP is often associated with TE, TE can manifest outside the menstrual period too, leading to pneumothorax. This lesser-known and often misunderstood condition is referred to as thoracic endometriosis-related non-catamenial pneumothorax (TER non-CP) [4, 5].

Assessing its incidence presents even more challenges due to underdiagnosis and various biases of the studies involving premenopausal women with SP, such as the exclusion of non-operated cases, diversity in investigative approaches, and a lack of pathological evidence.

The precise pathophysiological mechanisms underlying CP are not fully elucidated and various theories have been proposed: retrograde migration; endometrial tissue implantation; diaphragmatic defects; and hormonal fluctuations [6–16].

While a unanimous consensus on the best therapeutic management for CP is still lacking, there is a widely accepted recommendation of early surgical intervention, with a preference for a video-assisted thoracic surgery (VATS) approach for an accurate pleural cavity exploration [17, 18].

The surgery usually consists of the resection of endometrial foci, the repair of diaphragmatic defects (stapling/resection/prosthetic replacement), accurate pleurodesis and subsequent hormonal therapy. Since there is no according comparison in the literature, choosing among the variety of options is often a personal choice, based on the single surgeon’s experience. Therefore, we decided to conduct our study to delineate the most appropriate and effective surgical treatment, comparing the expertise of five European centres.

## MATERIALS AND METHODS

We conducted a European multicentre retrospective cohort study revising clinical and pathological data from March 2009 to December 2022. The participant centres were: (i) Careggi University Hospital (Florence, Italy)—Coordinator centre; (ii) University Hospital of Pisa (Pisa, Italy); (iii) Salamanca University Hospital (Salamanca, Spain); (iv) Sheffield Teaching Hospitals (Sheffield, UK); and (v) Bari University Hospital (Bari, Italy).

We evaluated all the cases of SP occurring in women of childbearing age with histological diagnosis of thoracic endometriosis or intraoperative evidence of diaphragmatic or pleural endometrial foci.

CP was defined as presenting between the day before and up to 72 h after the onset of menstruation. Endometriosis-related non-catamenial pneumothorax cases were included as well.

Our Department Internal Review Board granted approval and waived the requirement for specific informed consent and database monitoring for this retrospective analysis since all patients’ data were deidentified prior to analysis and publication (committee minute number: 3150–23).

We collected data about age, side, previous diagnosis of endometriosis, and concomitant abdominal symptoms. We then analysed the details of the surgical procedures, focusing on the evidence of diaphragmatic defects and their treatment, the length of the hospitalization, the postoperative hormonal treatment and relapses.

### Subdivision of patients into groups

We decide to group our patients based on the diaphragmatic treatment received, to evaluate the best surgical option.

We focused our statistical analyses firstly on comparing the patients who received prosthetic replacement of the diaphragm (Group A) and the ones who did not (direct diaphragmatic suture, stapling or no treatment—Group B).

Secondly, we decided to remove from the no-prosthesis group patients who did not receive any diaphragmatic treatment and to analyse only the patients who underwent diaphragmatic surgery, comparing prosthesis with conservative treatment, i.e. patients with direct suture or stapling of the diaphragm (Group C).

We finally compared the perioperative data of patients who received the synthetic mesh with the ones with the biological prosthesis.

Follow-up was updated in June 2023 by telephonic interview.

### Statistical analysis

Statistical analysis was performed using SPSS 25.0 (SPSS Inc., Chicago, IL, USA). Standard descriptive statistics have been used to summarize data, with respect to demographic and clinical characteristics. Continuous variables, expressed as median value and IQR, were compared by the Mann–Whitney *U*-test; categorical variables were analysed with Fisher’s exact test; follow-ups have been compared by unpaired *t*-test of median. *P*-values below 0.05 were considered as statistically significant. To define predictors for recurrence, a univariate exact logistic regression analysis was performed for clinical and surgical treatment variables that may influence recurrence.

Finally, we analysed the recurrences between the groups using the reverse Kaplan–Meier method.

## RESULTS

Thirty-six patients were included in this study. Clinical data and association with endometriosis, concurrent hormonal therapy and previous abdominal history are depicted in Table 1. Surgical approaches and conversion rates are shown in Table 2.

**Table 1:** Preclinical data

Age	35 years (range 23–48)
Right side	31 pts (86.1%)
Catamenial	30 pts (83.3%)
Previous diagnosis of endometriosis	10 pts (27.8%)
Abdominal symptoms	7 pts (19.4%)
Previous hormonal treatment	5 pts (13.9%)
No abdominal history	14 pts (38.9%)

pts = patients.

**Table 2:** Surgical approaches

	Surgical approach	
	VATS	Open
Number of patients	34 (94.4%) ↓ 9 (26.5%) converted to open	2 (5.6%)

### Patient assessment and surgical techniques

**Figure 1:**
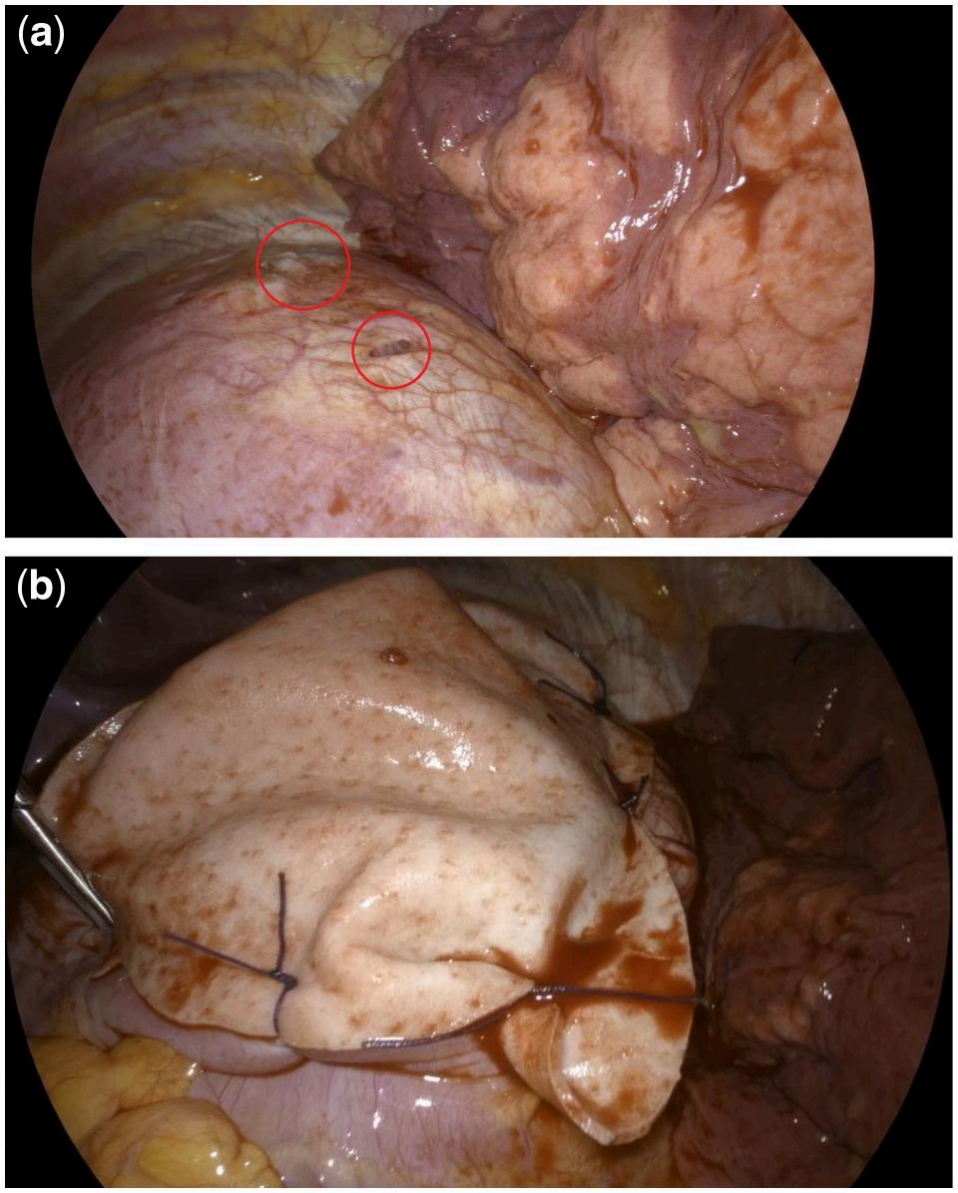
(**a**) Diaphragmatic alterations (red circles). (**b**) Diaphragm covering with biological mesh (Permacol).

During the surgical procedure, 30 patients (83.3%) were confirmed with diaphragmatic involvement and different strategies have been adopted to repair the fenestrations (Fig. 1a). In 4 (11.1%) patients, direct suture was performed, and stapling in 2 (5.6%).

Eleven patients (30.6%) did not undergo diaphragmatic surgery. Five of them (13.9%), even if with diaphragmatic alterations did not receive any diaphragmatic surgery according to the surgeon’s expertise. Six (16.7%) patients showed preserved diaphragm with no lesions instead.

None of the patients had pleural endometrial foci or alterations. Moreover, 19 of them got prosthetic replacement of the defect, 14 (38.9%) with synthetic graft and 5 (13.9%) with biological mesh (cross-linked porcine dermal collagen mesh—Permacol) (Fig. 1b).

Since these choices have been made according to the centre and surgeon’s expertise we can see quite a treatment variability, including the type of pleurodesis: 6 (16.7%) patients received mechanical pleurodesis (5 abrasions, 1 floating ball), 15 (41.6%) talc insufflation, 3 (8.3%) pleurectomies and 12 (33.3%) a combination of these methods.

### Perioperative variables

A median of 1.5 (IQR 1) chest drainages per patient have been used and they have been removed after a median of 5 (IQR 2) days. Patients were discharged after a median of 6 (IQR 3)  days.

Median follow-up was 54 months (IQR 82). During this period, 15 (41.7%) recurrences occurred: 5 patients who received the synthetic mesh; 7 who had pleurodesis alone; and 3 who underwent direct suture of the diaphragm. None of the patients who received the biological mesh presented recurrences during the follow-up period (Table 3).

**Table 3:** Treatments and recurrences percentages

Diaphragm repair		Treatment		Recurrences
Yes	25 (69.4%)	Synthetic mesh	14 (56%)	5 (35.7%)
		Biological mesh	5 (20%)	0 (0%)
		Suture	4 (16%)	3 (50%)
		Stapler	2 (8%)	
No	11 (30.6%)	No evidence	6 (54.5%)	7 (63%)
		Not performed	5 (45.5%)	

In addition, 26 (72.2%) patients received postoperative hormonal blockade and 1 patient started physiological menopause 1 month after surgery. Due to the small sample size, we decided to depict all the perioperative details in Table 4 for a more accurate evaluation.

**Table 4:** Perioperative data

Pt ID	VATS/open	Number of VATS ports	Conversion	Diaphr defects	Diaphr surgery	Pleurodesis	Number of chest drains	Recurrences	Postop hormonal therapy
1	Open			Yes	BioMesh	Pleurectomy	2	No	Menopause
2	VATS	2	No	Yes	None	Talc	1	Yes	Yes
3	VATS	2	No	Yes	None	Talc	1	Yes	Yes
4	VATS	2	No	Yes	BioMesh	Talc	1	No	Yes
5	VATS	2	No	Yes	BioMesh	Abr + Talc	2	No	Yes
6	VATS	2	No	Yes	BioMesh	Abr	1	No	Yes
7	VATS	1	Yes	Yes	BioMesh	Talc	1	No	Yes
8	VATS	2	No	No	None	Floating Ball	2	No	No
9	VATS	2	No	Yes	None	Abr	2	Yes	No
10	VATS	3	No	No	None	Abr	1	No	Yes
11	VATS	2	No	Yes	None	Abr + Talc	1	Yes	Yes
12	VATS	2	No	No	None	Talc	2	No	Yes
13	VATS	1	No	No	None	Talc	1	Yes	Yes
14	VATS	3	No	Yes	None	Talc	1	Yes	Yes
15	VATS	2	No	No	None	Abr	1	No	Yes
16	VATS	3	No	Yes	SynMesh	Talc	2	No	Yes
17	VATS	3	No	Yes	SynMesh	Talc	2	Yes	Yes
18	VATS	3	No	Yes	SynMesh	Talc	2	No	Yes
19	VATS	2	No	Yes	SynMesh	Talc	2	Yes	Yes
20	VATS	2	Yes	Yes	SynMesh	Pleurectomy	1	Yes	Yes
21	VATS	2	No	Yes	SynMesh	Abr + Talc	1	No	No
22	VATS	2	No	Yes	SynMesh	Abr + Talc	1	No	No
23	VATS	2	No	Yes	SynMesh	Abr + Talc	1	No	Yes
24	VATS	2	No	Yes	SynMesh	Abr + Talc	1	Yes	Yes
25	VATS	2	No	Yes	SynMesh	Abr + Talc	1	No	No
26	VATS	2	Yes	Yes	SynMesh	Pleurectomy	2	No	Yes
27	VATS	2	No	Yes	SynMesh	Talc	1	No	Yes
28	VATS	2	No	Yes	SynMesh	Talc	1	No	Yes
29	VATS	1	Yes	Yes	Suture	Abr + Talc	2	Yes	Yes
30	Open			Yes	Suture	Talc	2	Yes	No
31	VATS	1	Yes	Yes	Stapler	Abr + Talc	2	No	No
32	VATS	1	Yes	Yes	SynMesh	Abr	3	Yes	No
33	VATS	2	Yes	Yes	Suture	Talc	2	Yes	Yes
34	VATS	3	Yes	Yes	Stapler	Abr + Talc	2	No	Yes
35	VATS	1	Yes	Yes	Suture	Abr + Talc	2	No	No
36	VATS	3	No	No	None	Abr + Talc	2	Yes	Yes

Abr, abrasion; BioMesh, biological mesh; Diaphr, diaphragmatic; Postop, postoperative; SynMesh, synthetic mesh.

In the univariate analysis assessing the impact of postoperative medical therapy, our data showed a non-significant association [odds ratio (OR) = 0.583, 95% CI 0.119–2.849, *P* = 0.505], without indicating a clear trend, despite the already widely accepted indication to add hormonal therapy to these patients. Comparisons between the groups are shown in Table 5.

**Table 5:** Comparison between the groups

	Group A	Group B	*P*
	Prosthesis (*n* = 19)	No prosthesis (*n* = 17)	
Age (years)	33 (14)	37 (10)	0.325
Conversions	4 (21%)	5 (29.4%)	0.765
Drains removal (days)	5 (3)	5 (2)	0.802
Hospital stay (days)	6 (4)	7 (4)	0.098
Recurrences	5 (26.3%)	10 (56.8%)	0.102
Median follow-up (months)	87 (102)	44 (48)	0.182

	Group A	Group C	*P*
	Prosthesis (*n* = 19)	Suture/Stapler (*n* = 6)	
Age (years)	33 (14)	42 (13)	0.073
Conversions	4 (21%)	5 + 1 (100%)	0.003
Drains removal (days)	5(3)	4 (3)	1
Hospital stay (days)	6 (4)	7 (4)	0.175
Recurrences	5 (26.3%)	3 (50%)	0.344
Median follow-up (months)	87 (102)	34 (28)	0.160

	Synthetic mesh (*n* = 14)	Biological mesh (*n* = 5)	*P*
Age (years)	32 (7)	44 (12)	0.141
Conversions	3 (21.4%)	1 (20%)	1
Drains removal (days)	5 (2)	6 (5)	0.262
Hospital stay (days)	5.5 (2)	8 (5)	0.262
Recurrences	5 (35.7%)	0 (0%)	0.257
Median follow-up (months)	101.5 (95)	46 (72)	0.303

### Comparison between Group A and Group B

Every patient in the prosthetic group had confirmation of diaphragmatic fenestrations before the mesh implant.

The median age was 33 (IQR 14) years for the first group and 37 (IQR 10) years for the second group, with no statistically significant difference (*P* = 0.325).

The surgical approach was VATS for 18 (94.7%) patients in Group A and 16 (94.1%) in Group B. In the first group, four patients were converted to thoracotomy, and five in the second group, with no significant difference (*P* = 0.765).

In Group A, chest drains were removed after a median of 5 days (IQR 3) and patients discharged after a median of 6 days (IQR 4). In Group B drains were removed after a median of 5 days (IQR 2) and were discharged after a median of 7 days (IQR 4). In neither case did a statistical difference occur (*P* = 0.802 and *P* = 0.098, respectively).

In Group A, recurrence occurred in 5 patients (26.3%) versus 10 (58.8%) in Group B, and comparative analyses showed *P* = 0.102. Moreover, at univariate analyses, OR was 0.250 (95% CI 0.061–1.019) for Group A with *P* = 0.053.

Follow-up lasted for 44 months (IQR 48) for the group without prosthesis and 87 (IQR 102) for those with prosthesis (*P* = 0.182).

**Figure 2:**
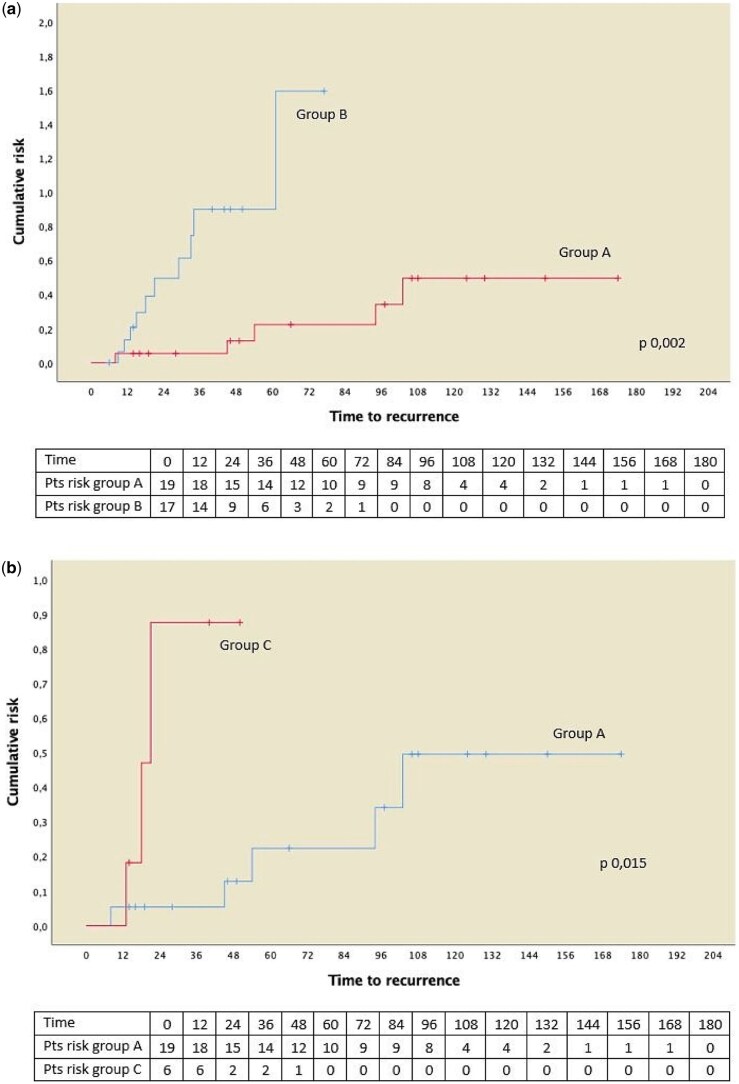
(**a**) Reverse Kaplan–Meier for recurrence risk between use of prosthesis during surgical procedure (Group A) versus no prosthetic treatment (Group B). (**b**) Reverse Kaplan–Meier for recurrence risk between use of prosthesis during surgical procedure (Group A) versus other diaphragmatic treatment (Group C).

The analysis of the recurrences between the groups tested via the reverse Kaplan–Meier method are depicted in Fig. 2b and show a significant difference (*P* = 0.002) in favour of the prosthetic group.

### Comparison between Group A and Group C

Chest tubes were removed after a comparable median of days (*P* = 1), 4 days (IQR 3) in Group C and 5 days (IQR 3) in Group A. Length of hospitalization was also similar: 7 (IQR 4) versus 6 (4) days, *P* = 0.175.

Notably, Group C showed a 100% conversion rate versus 21% of Group A, which was statistically significant (*P* = 0.003).

Recurrences occurred in three patients out of six in Group C (50%) and in five in Group A (26.3%) showing, even if without statistical significance, a slight numerical tendency in favour of Group A (*P* = 0.344).

The linear regression analysis in this case showed an OR of 2.800 (95% CI 0.419–18.689; *P* = 0.288).

Follow-up was 34 (IQR 28) versus 87 (IQR 102) months (*P* = 0.160).

**Figure 3:**
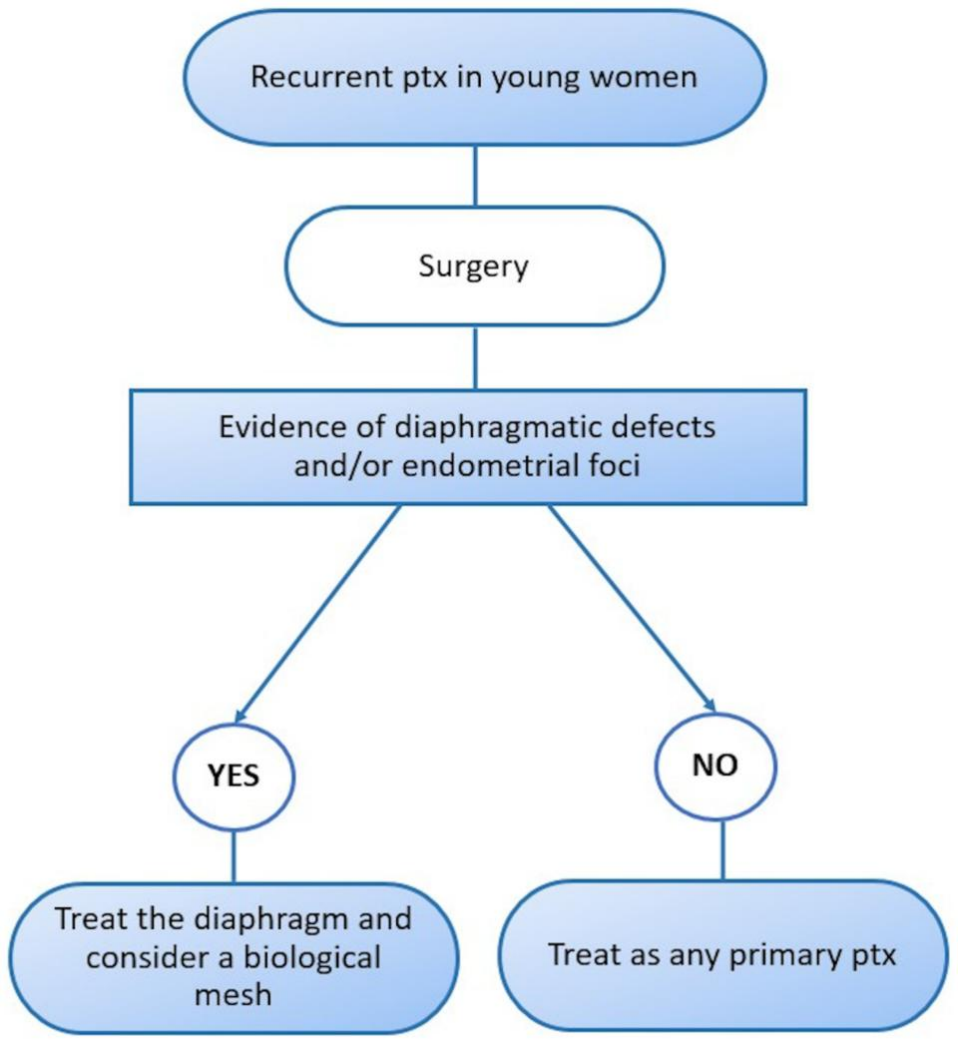
Treatment flowchart. ptx, pneumothorax.

The analysis of the recurrences between the groups tested via the reverse Kaplan–Meier method is depicted in Fig. 3 and shows a significant difference (*P* = 0.0015) in favour of the prosthetic group.

### Comparison between the synthetic and biological meshes

We observed three conversions in the first group compared with one conversion in the biological prosthesis group (*P* = 1).

Both the removal of the chest tubes [5 (IQR 2) and 6 (IQR 5)] and the length of hospital stay [5.5 (IQR 2) and 8 (IQR 5)] resulted in overlapping without a statistically significant difference (*P* = 0.262; *P* = 0.262, respectively).

We finally compared the recurrences of patients who received the synthetic mesh with the ones with the biological prosthesis: 5 versus 0, respectively (*P* = 0.257).

Median follow-up of the synthetic mesh was 101.5 (IQR 95) and 46 months (IQR 72) in the biological group (*P* = 0.303).

## DISCUSSION

CP is an uncommon condition and its precise prevalence is still difficult to assess due to underdiagnosis and under-reporting.

However, the most recent available data suggest that it accounts for a significant proportion of SP cases in women of childbearing age compared with what was believed in past years [3, 16, 19].

The first, and to date only, prospective study on CP was conducted by Alifano and colleagues [3] and highlighted how frequently CP results when attention is given to investigating the temporal relationship between pneumothorax and menses, and an accurate VATS inspection of the diaphragm is conducted.

In fact, while the previous studies reported CP prevalence between 0.9% and 5.6% [16, 19] among all the SP in women, the prospective study reported that 25% of SP in women referring for surgery were actually CP cases (8 cases among 32 women in 18 months).

Although these numbers may appear low, they represent a group of young women suffering from years of pain and discomfort, with recurrent hospital stays and a strong impact on their quality of life.

Data about the recurrence of CP vary from 8% to 40% of patients despite the combined approach of surgery and hormonal suppression, and these rates are significantly higher than those of surgically treated patients with primary SP (<5%) [4].

The most effective approach results in a multimodal treatment of surgery, pleurodesis and postoperative hormonal therapy, and several retrospective studies have been dedicated to analysing the optimal surgical treatment options [20–24]. The importance of addressing the diaphragmatic defects is now well established even with a wide variety of surgical options. The heterogeneity of treatments for CP reflects the complexity of this condition and a one-size-fits-all solution is still lacking. For instance, Koike and colleagues exhibit good outcomes in two patients treated with diaphragmatic and pleural covering with cellulose [20].

A more consistent number of retrospective studies show a trend of preference for the prosthetic coverage of the diaphragm.

Campisi and colleagues underlined the importance of diaphragmatic surgery combined with pleurodesis and postoperative hormonal therapy to reduce the recurrence rates [21]. In their retrospective study they showed a 100% recurrence rate in the pleurodesis group versus only 12.5% in the patients treated with diaphragmatic surgery. The best outcome with no recurrences or chest pain was obtained with the three patients who received a polyglactin mesh. The authors conclude by suggesting wide use of the mesh insertion, even when there are not clear diaphragmatic lesions.

Indeed, sometimes diaphragmatic fenestrations can be millimetric and challenging to diagnose, both in VATS and in thoracotomy.

Additionally, suturing or stapling the diaphragm, especially in cases of numerous and large fenestrations or nodules, can significantly reduce its respiratory functionality and increase the risk of lacerations.

In a retrospective case series by Attaran and colleagues, 11 out of 12 patients with CP underwent VATS pleurectomy and diaphragmatic repair with prosthetic placement of polytetrafluoroethylene mesh, followed by postoperative hormonal suppressive therapy, and the reported rates of recurrence are very low (1%) after a median follow-up of 45.8 months [22].

The patients treated by Leong and colleagues also underwent diaphragmatic prosthesis placement (polyglactin or polypropylene) with only one recurrence [23].

Moreover, the series presented by Bagan and colleagues showed a significant difference (*P* = 0.0016) between patients treated with diaphragmatic coverage by a polyglactin mesh versus pleurodesis alone [24].

Consistent with these results, our data, although not reaching statistical significance, demonstrate the effectiveness and safety of performing diaphragmatic prosthesis placement upfront.

In fact, in none of the analyses we conducted, did patients who underwent prosthesis placement demonstrate a longer hospitalization, and early postoperative outcomes have been overlapping. Furthermore, even with no statistical significance, the data exhibited a numerical difference, which might be clinically relevant regarding the slightly longer hospital stay in patients who underwent conservative diaphragmatic treatment (*P* = 0.084), despite a similar postoperative day of chest tube removal. However, it’s worth noting that this finding could be attributed to the fact that ours is a multicentre study and thus it reflects varied postoperative managements.

It is also worth highlighting the significant difference in the conversion rate when compared with the patients who underwent direct suturing or stapling of the diaphragm (100% vs 22.2%, *P* = 0.004). This could suggest that such a surgical procedure can often be complex and challenging and, at times, less effective.

Furthermore, when we compare the recurrence rates, we observe that although not statistically significant, the group of patients who underwent prosthesis placement tend to show a better outcome (*P* = 0.09 and *P* = 0.344), underlying the importance of always performing diaphragmatic surgery for these patients.

Moreover, performing the survival analysis with the reverse Kaplan–Meier method, our data highlighted a significant statistical difference in favour of the prosthetic placement compared both with the non-prosthetic group (*P* = 0.002) and the other treatments of the diaphragm (*P* = 0.0015).

Finally, comparing the recurrences between the two groups of prosthesis placement, it is true that we did not find a statistically significant difference (*P* = 0.257), but it is also true that our experience shows 0% recurrences in patients with biological mesh. We certainly need further data and a longer follow-up, since the use of biological mesh is a more recent surgical practice, but the trend we observed could suggest a continuation down this path.

In the end, we can say that our series confirms the international trend shown by the paper published by Pathak *et al.* [25]. Their research about the best evidence treatment for CP concluded that this disease, to obtain a significant reduction of recurrences, requires a multimodality approach. They highlighted the importance of performing a combination of diaphragmatic repair, pleurodesis and postoperative hormonal treatment.

This review also stresses the plural biases that the whole literature presents about CP, in fact every article presents little samples with heterogeneity of surgical and medical treatment. For example, we decided not to perform any statistical analysis regarding the pleurodesis techniques since there is no current evidence on the superiority of one specific method and every centre decided to perform it according to their experience.

### Study limitations

As a retrospective multicentre study we are aware of the multiple biases of our data related to the comparison between heterogeneous patients and different treatments in such a small sample.

Moreover, focusing on the in-hospital variables and surgical techniques, the epidemiological and physiological data, which contribute to the considerable heterogeneity of the study population, were not adequately analysed, nor was the management and timing of the recurrent cases.

## CONCLUSIONS

In conclusion, this study aims to provide an in-depth exploration of surgical treatment of CP, summarizing the current state of knowledge on this complex disorder.

Due to the overlap of symptoms with more common conditions, many healthcare professionals may not consider CP as a potential diagnosis, leading to delayed or misdiagnosis. Increased awareness and more research efforts are needed to ensure that women with CP receive prompt and accurate diagnosis and appropriate treatment, ultimately improving their well-being.

Our experience suggests an early surgical approach, with thorough attention to identify and to treat all the diaphragmatic defects. In particular, prosthetic replacement resulted in a safe and effective procedure and our data also suggest that biological mesh should be preferred in this setting, showing excellent postoperative and long-term results.

Well aware of our study’s limitations, we decided to design a flowchart for the treatment of CP to provide a clear, structured approach for clinicians in managing this rare condition (Fig. 2a). By creating a visual guide, we hope to ease the decision-making process, ensuring prompt and accurate treatment. Additionally, it could serve as a useful tool for both training and clinical practice, enhancing patient outcomes.

However, the true prevalence of TE and CP is still underestimated and a unanimous consensus on the best therapeutic pathway is still lacking, highlighting the need for dedicated studies and increased awareness in the medical community.

## Data Availability

The data underlying this article are available in the article.

## ABBREVIATIONS

CI: Confidence interval
CP: Catamenial pneumothorax
IQR: Interquartile range
OR: Odds ratio
SP: Spontaneous pneumothorax
TE: Thoracic endometriosis
VATS: Video-assisted thoracic surgery
